# Anti-retroviral innate immune responses in multiple lymphoid tissues elicited by protective live attenuated SIV vaccination

**DOI:** 10.1186/1742-4690-8-S2-O29

**Published:** 2011-10-03

**Authors:** Giada Mattiuzzo, Claire Ham, Deborah Ferguson, Nicola Rose, Martin Cranage, Neil Almond, Greg Towers, Neil Berry

**Affiliations:** 1 HPA-NIBSC, Division of Retrovirology, Potters Bar, Herts, UK; 2 St George's, University of London, Centre for Infection and Immunity, London, UK; 3 MRC Centre for Medical Molecular Virology, Division of Infection and Immunity, University College London, Cruciform Building, London, UK

## Background

Vaccination with live attenuated SIV confers potent protection against wild-type challenge, although correlates of adaptive immunity have not been reliably demonstrated. Despite safety concerns for this vaccine approach in humans, understanding the mechanism of this protection may inform and guide HIV vaccine design. In Mauritian-origin cynomolgus macaques (*Macaca fascicularis*), we have demonstrated early, potent protection against the heterologous SIVsmE660 virus stock following vaccination with the minimally attenuated SIVmac251/C8 vaccine [1]. Failure to associate correlates of protection with adaptive immune responses and the emergence of protection as early as 3 weeks post vaccination has led us to characterise anti-retroviral innate immune responses in this model system.

## Materials and methods

The distribution and level of intracellular virus replication in multiple lymphoid tissues including the small and large intestine, spleen, inferior and superior mesenteric lymph nodes (i and sMLN) was determined by quantitative PCR (qPCR) over a time-course of 3, 7, 10, 21 and 125 days following vaccination with live attenuated SIVmacC8. Virus presence in the same tissues was also confirmed by *in-situ* hybridisation. To evaluate aspects of the innate immune response, TRIM5α genotype was determined and transcriptome analysis performed by qPCR for multiple ínterferon-inducing and induced genes including IRF-7, STAT-1, TRIM5α, ApoBEC3G, tetherin, TRIM-22. In addition, a range of immunological markers of cell-surface protein expression were measured by immunohistochemistry.

## Results

Following SIV inoculation, rapid and widespread dissemination of virus was identified in multiple lymphoid tissues. The MLN represented the site with the most consistent frequency of virus infection, with virus detected at 3 days post-infection. Transcriptome analysis indicated a significant up-regulation of TRIM5α, ApoBEC3G and IRF-7 with the highest levels co-inciding with the peak of primary viraemia at day 10. Subsequently, responses tended to follow the control of acute-phase replication beyond day 21 which remained above baseline levels at day 125. Retrospective analysis of protective vaccine studies indicates raised cell-surface markers of mediators of innate immunity including CD68+ macrophages, and S100 and DC-SIGN on dendritic cells which were upregulated and maintained in vaccinated, protected macaques but diminished in cases of retroviral superinfection.

## Conclusions

These data suggest a broad anti-retroviral innate response generated during primary SIV infection, driven in this instance by live retrovirus vaccination with a *nef*-disrupted virus. These appear to be playing a role in controlling the vaccine virus, co-incident with widespread changes in cell-population dynamics previously described for this vaccine [2] which are maintained above basal levels by a persistent virus replicating in multiple lymphoid tissues. The relative contribution of these responses in vaccine protection is being investigated.
